# When Appearances Deceive: A Silent Somatotroph Macroadenoma Presenting With Acromegaloid Features and Paradoxically Low Insulin-Like Growth Factor-1

**DOI:** 10.7759/cureus.118088

**Published:** 2026-10-18

**Authors:** Emily M Mayar, Sing Y Sim

**Affiliations:** 1 Endocrinology, University Hospitals Sussex NHS Foundation Trust, Brighton, GBR

**Keywords:** acromegaloid features, insulin-like growth factor-1 (igf-1), pituitary neuroendocrine tumour, silent somatotroph adenoma, stalk effect, transsphenoidal surgery

## Abstract

Silent somatotroph adenomas are uncommon pituitary neuroendocrine tumours (PitNETs) of the PIT1 lineage that express growth hormone (GH) immunohistochemically but without biochemical evidence of acromegaly. We report the case of a 32-year-old male patient referred with recurrent epistaxis, headache, right-sided diplopia, and third cranial nerve palsy. Clinical examination revealed acromegaloid features including frontal bossing, spade-like hands, and coarse facies, which were strongly suggestive of longstanding GH excess. However, preoperative insulin-like growth factor-1 (IGF-1) was paradoxically low at 13.1 nmol/L (reference range 15.5-33.1 nmol/L), creating a striking clinical-biochemical discordance. MRI demonstrated a giant sellar and suprasellar mass with chiasmal compression, right cavernous sinus invasion, and extension into the paranasal sinuses. The patient underwent endoscopic transsphenoidal resection. Neuropathology confirmed a densely granulated PIT1-lineage somatotroph adenoma with scattered GH positivity, diffuse Pit-1 staining, and a Ki-67 index below 2%. This case highlights the diagnostic challenge posed by silent somatotroph adenomas, underscores the limitations of biochemical assessment alone, and reinforces the essential role of histopathology and immunohistochemistry in establishing a definitive diagnosis. Long-term endocrine and neurosurgical surveillance is strongly recommended given the potential for phenotypic transformation to a functioning acromegaly syndrome.

## Introduction

Pituitary neuroendocrine tumours (PitNETs) account for approximately 10-20% of all intracranial neoplasms [1]. The 2022 World Health Organization (WHO) classification characterises PitNETs according to cell lineage, defined by immunohistochemical expression of lineage-specific transcription factors: PIT1 for the somatotroph, lactotroph, and thyrotroph lineages; TPIT for the corticotroph lineage; and SF1 for the gonadotroph lineage [1,2]. This lineage-based framework supersedes earlier morphological classification schemes and has direct implications for tumour behaviour, surveillance, and management.

Silent somatotroph adenomas are PIT1-lineage PitNETs that are immunohistochemically positive for growth hormone (GH) yet produce no clinical syndrome of GH excess. They account for approximately 2-4% of surgically excised pituitary adenomas and are most commonly identified incidentally or via mass-effect symptoms such as headache or visual disturbance [1,3]. Silent somatotroph adenomas predominantly affect younger women, carry a significant risk of recurrence, and frequently require adjuvant radiotherapy, with one large series reporting an 82% female preponderance. Silent somatotroph adenomas carry a risk of biochemical progression over time, with elevated insulin-like growth factor-1 (IGF-1) documented in approximately 11% of cases in one large series, necessitating long-term endocrine follow-up [3]. 

Although most patients with acromegaly demonstrate high circulating IGF-1 levels, discordance between clinical phenotype and biochemical assessment is occasionally seen; this presents a diagnostic challenge as acromegaloid phenotypic features may reflect previous GH excess, non-GH-related acromegaloid syndrome, or very rarely a GH-immunoreactive PitNET with limited secretory activity. Whilst a low or normal IGF-1 may raise suspicion of biochemical silence, formal exclusion of GH excess requires an oral glucose tolerance test (OGTT) with GH suppression; IGF-1 alone is insufficient to establish or exclude active acromegaly [4]. In this context, definitive diagnosis requires histopathological confirmation with GH and transcription factor immunostaining [1]. Here, we report such a case, presenting clinical, radiological, and histopathological data.

This case was previously presented as a poster at the European Congress of Endocrinology in Prague, Czech Republic, in May 2026.

## Case presentation

A 32-year-old male patient with no significant past medical history was referred to the endocrinology outpatient department following recurrent epistaxis, persistent headache, right-sided diplopia, and third cranial nerve palsy. On examination, the patient displayed a constellation of acromegaloid features: frontal bossing, spade-like hands, and coarse facial characteristics, clinically suggestive of longstanding GH excess. Notably, he reported no hyperhidrosis and no rapid weight gain.

Ophthalmological assessment demonstrated mild enlargement of the right blind spot with preserved visual fields. MRI of the pituitary demonstrated a large sellar and suprasellar mass (4.5 cm x 4 cm) with 37.9 mm suprasellar extension, chiasmal compression, bilateral cavernous sinus invasion (right greater than left), and extension into the sphenoid sinus, clivus, and right superior orbital fissure. An initial nasal tissue biopsy had been performed to exclude nasopharyngeal malignancy; histology revealed only benign adenoid tissue.

Preoperative biochemistry demonstrated markedly elevated prolactin at 4107 mIU/L (reference range 86-324 mIU/L), consistent with stalk-effect hyperprolactinaemia secondary to the large sellar mass [5]. Crucially, IGF-1 was paradoxically low at 13.1 nmol/L (reference range 15.5-33.1 nmol/L), creating a striking discordance between the clinical acromegaloid phenotype and the biochemical profile. The short synacthen test was normal. Full anterior pituitary hormone assessment demonstrated borderline-low total testosterone at 5.4 nmol/L (reference range 8.6-29 nmol/L), in keeping with hypogonadotrophic hypogonadism secondary to mass effect (Table 1).

**Table 1:** Preoperative biochemistry demonstrating paradoxically low IGF-1 in the context of acromegaloid clinical features and stalk-effect hyperprolactinaemia. IGF-1: insulin-like growth factor-1; TSH: thyroid stimulating hormone; FSH: follicle-stimulating hormone; LH: luteinizing hormone

Parameter	Patient Value	Reference Range
IGF-1	13.1 nmol/L	15.5–33.1 nmol/L
Prolactin	4107 mIU/L	86–324 mIU/L
Free Testosterone	158 pmol/L	198–619 pmol/L
TSH	1.37 mU/L	0.27–4.2 mU/L
Free T4	15.6 pmol/L	11–22 pmol/L
FSH	3.8 IU/L	1.5–12.4 IU/L
LH	4.1 IU/L	1.7–8.6 IU/L
Short Synacthen Test	Normal. 0 minutes: 226 nmol/L. 30 minutes: 498 nmol/L	—

Random serum GH concentration and OGTT with GH suppression were not performed prior to surgery, and repeat IGF-1 measurement was not feasible, as urgent neurosurgical intervention was indicated owing to his progressive cranial nerve palsy and optic pathway compression. Given the markedly elevated pre-operative prolactin, cabergoline 500 μg weekly was initiated empirically pending surgical planning. This achieved suppression of prolactin; however, levels rose to 3844 mIU/L following discontinuation, and cabergoline was recommenced at the same dose. 

The patient subsequently underwent endoscopic transsphenoidal resection of the pituitary macroadenoma a year after the first presentation. Postoperatively, the patient developed transient diabetes insipidus managed with desmopressin (DDAVP), which resolved completely. A borderline postoperative cortisol of 178 nmol/L prompted precautionary temporary hydrocortisone replacement (15 mg morning and 5 mg afternoon). Visual function and residual anterior pituitary hormones were largely preserved.

Postoperative MRI confirmed debulking of the macroadenoma with a central surgical cavity, but identified residual tumour at multiple sites including the right cavernous sinus, clivus, suprasellar cistern, right anterior clinoid process, and right orbital fissures, with suprasellar nodules in contact with the left optic tract and optic chiasm (Figures 1, 2). 

**Figure 1:**
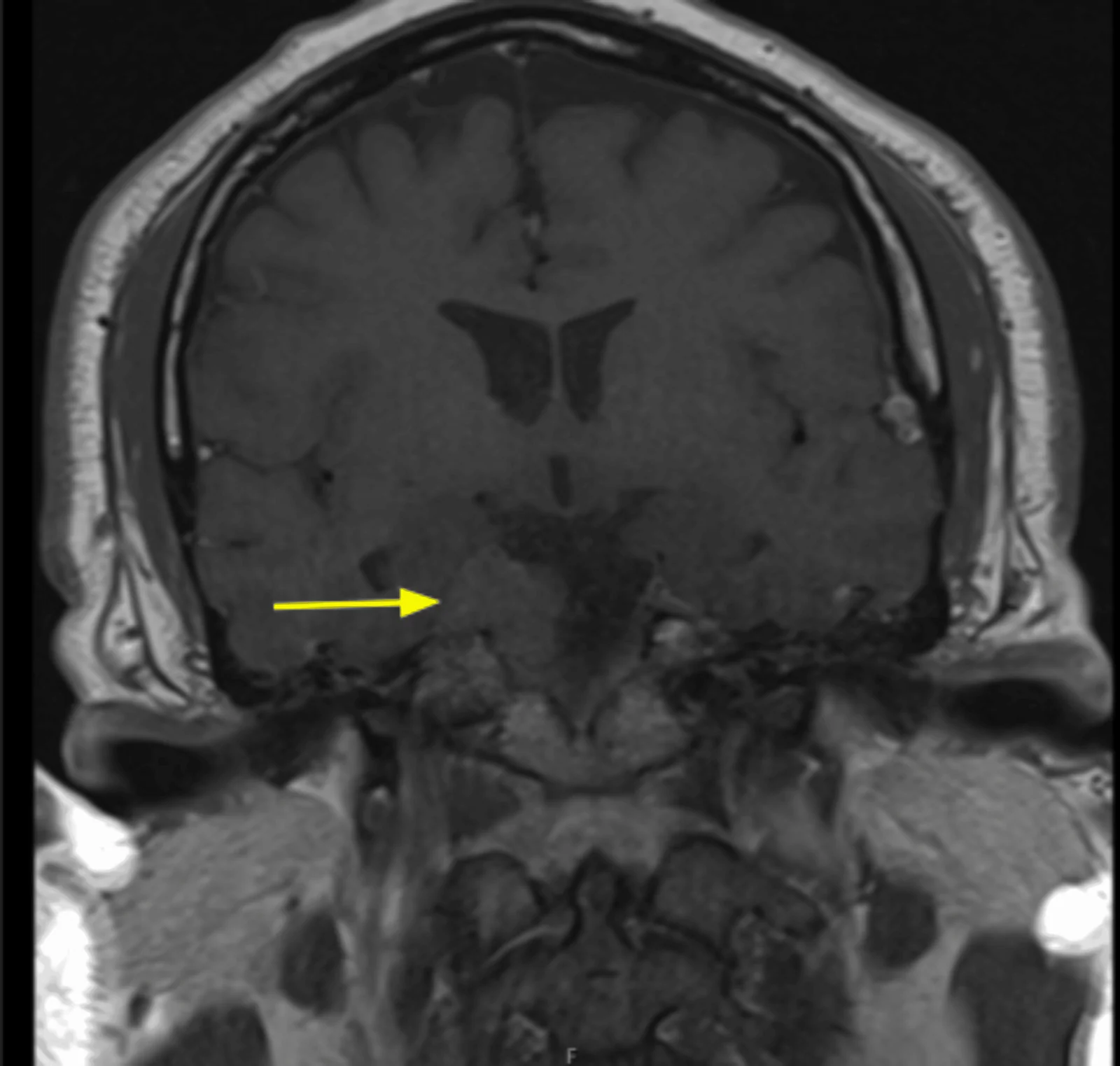
Coronal MRI demonstrating postoperative debulking of the pituitary macroadenoma with central surgical cavity and residual tumour in the right cavernous sinus and suprasellar region, with persistent nodules contacting the optic chiasm

**Figure 2:**
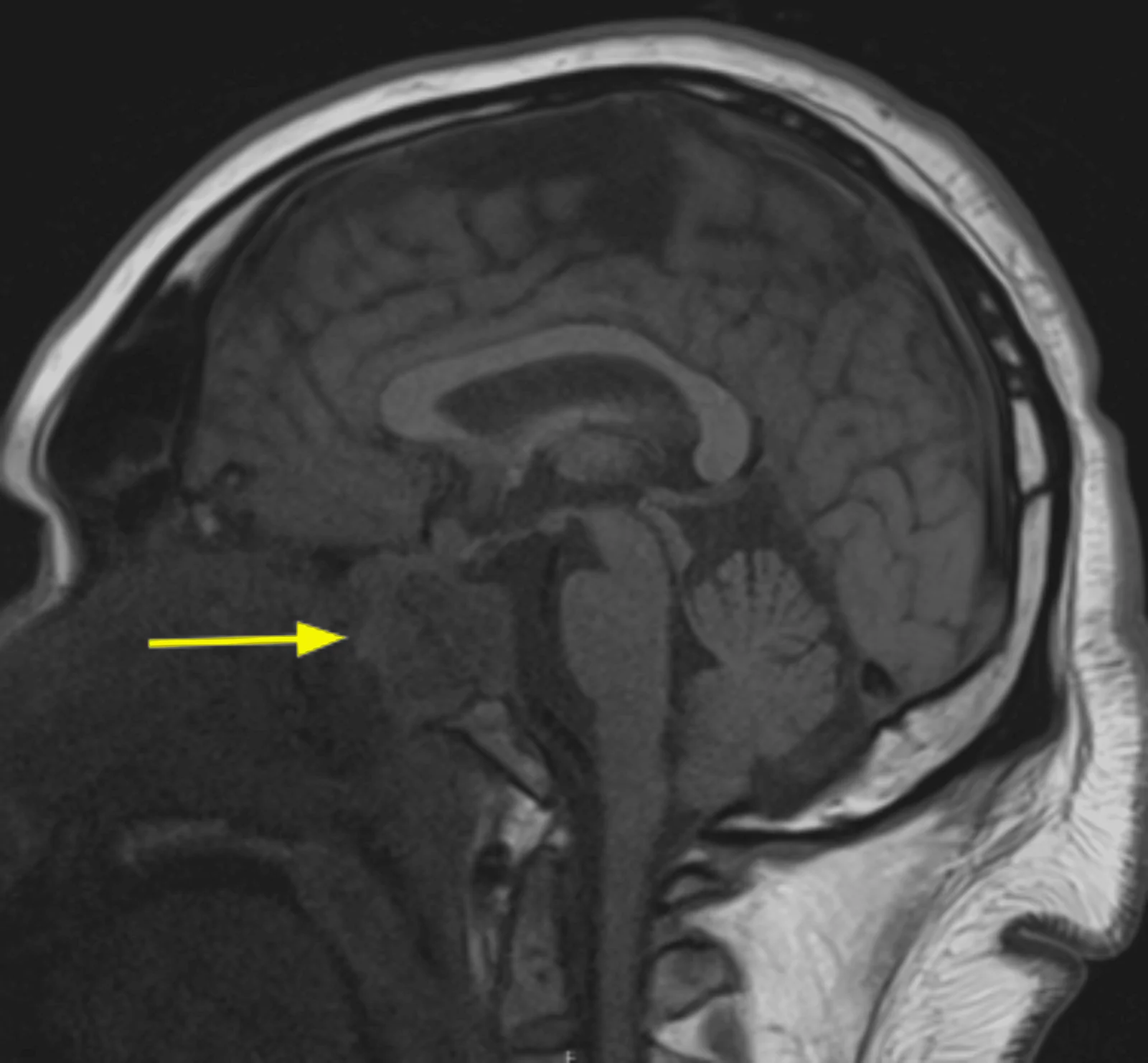
Sagittal MRI demonstrating postoperative cavity following transsphenoidal debulking, with residual tumour rind extending to the clivus and suprasellar cistern

The case was presented at the combined pituitary multidisciplinary team (MDT), and following discussion of the options, the patient opted for a conservative surveillance approach for the residual disease, with fractionated stereotactic radiotherapy available should progression occur. This is consistent with the known behaviour of silent somatotroph adenomas, in which 29% of patients require repeat surgery for recurrence and 18% undergo adjuvant radiotherapy in published series [3].

Histopathological examination of the resected specimen confirmed a PIT1-lineage somatotroph adenoma (densely granulated). Immunohistochemistry demonstrated scattered GH-positive tumour cells and diffuse, strong Pit-1 positivity. Staining for prolactin, adrenocorticotropic hormone (ACTH), luteinizing hormone (LH), follicle-stimulating hormone (FSH), thyroid-stimulating hormone (TSH), steroidogenic factor 1 (SF-1), and T-Pit was negative throughout. Ki-67 was low at less than 2%; however, a low Ki-67 index does not reliably exclude locally invasive clinical behaviour in PIT1-lineage tumours [6], as demonstrated in this case. Tumour architecture consisted of sheets, lobules, and trabeculae with effaced reticulin. No necrosis or invasion was identified. Diffuse cytokeratin staining without fibrous bodies confirmed the densely granulated subtype. He was discharged home eight days after the endoscopic transsphenoidal resection.

At the two-month follow-up after discharge, the patient reported subjective improvement in his sense of smell, taste, and singing voice (he is a professional opera singer). His diplopia had resolved, and ophthalmological review confirmed no new deterioration in visual function postoperatively. Hydrocortisone replacement was successfully discontinued following a normal short Synacthen test. He remains on cabergoline, reduced to 250 μg weekly, with his most recent prolactin level at 9 mIU/L, a year and four months after his surgery. MRI pituitary performed nine months postoperatively demonstrated stable appearance of the residual tumour. The patient remains under ongoing combined endocrine and neuroradiological surveillance.

## Discussion

This case illustrates an unusual clinicopathological discordance between convincing acromegaloid physical features, a low circulating IGF-1 concentration, and a GH-immunoreactive PIT1-lineage PitNET. This represents a classic diagnostic pitfall in pituitary disorders.

The mechanism underlying the dissociation between GH immunoreactivity and clinical/biochemical silence in these tumours remains poorly understood [1]; several hypotheses have been proposed, including defective post-translational processing, impaired GH secretion, and production of biologically inactive GH isoforms [7]. Despite clinical stigmata suggestive of chronic GH excess, the absence of symptoms of active hypersecretion, such as hyperhidrosis, together with inappropriately low IGF-1, is consistent with a silent somatotroph adenoma, a tumour that is biochemically and functionally quiescent despite GH immunopositivity on histology.

Definitive diagnosis requires histopathological assessment; biochemical profiling alone is insufficient. Immunohistochemistry should include GH to confirm somatotroph lineage and Pit-1 as the lineage-specific transcription factor [1]. The 2022 WHO classification framework endorses this transcription factor-based diagnostic approach [2]. In this case, histopathological assessment was essential in establishing the diagnosis, resolving the clinical-biochemical discordance, and confirming somatotroph lineage in the absence of biochemical GH excess. Classification of PitNETs by transcription factor lineage has direct implications for clinical behaviour and management: PIT1-lineage silent somatotroph adenomas are associated with a more aggressive course than silent gonadotroph adenomas, with higher recurrence rates and greater need for adjuvant radiotherapy [3]. The granulation pattern on cytokeratin staining further refines prognosis, with sparsely granulated somatotroph tumours demonstrating more aggressive behaviour than the densely granulated subtype identified in this case [1]. Furthermore, the Ki-67 proliferative index, whilst an imperfect predictor of invasiveness [6], as demonstrated in this case, remains a recommended component of histopathological assessment of silent pituitary adenomas [1].

When evaluating acromegaloid appearance with normal or low IGF-1, non-pituitary differentials should also be considered; these include pseudoacromegaly associated with severe insulin resistance, pachydermoperiostosis, acromegaloid facial appearance syndrome, or previously treated acromegaly [4]. The coexistence of a giant GH-immunoreactive PitNET made these diagnoses considerably less likely in this case. Instead, our patient’s marked acromegaloid stigmata may reflect years of tissue exposure to excess GH, suggesting that his tumour could have been biochemically active earlier in its development, though this remains speculative in the absence of historical GH or IGF-1 data.

The markedly elevated prolactin in this case is consistent with stalk-effect hyperprolactinaemia: compression or disruption of the pituitary stalk by an expanding sellar mass mechanically impairs dopamine delivery to the anterior pituitary, disinhibiting lactotrophs and elevating serum prolactin [5]. This phenomenon is recognised in approximately 40% of patients with non-functioning pituitary macroadenomas [8]. The markedly elevated prolactin, disproportionate to what would be expected from a non-secreting tumour, alongside the negative prolactin staining and absence of typical prolactinoma features, appropriately directed this case toward a stalk-effect aetiology rather than prolactinoma management. Careful interpretation of prolactin levels is essential in the context of large sellar masses; where hook effect is suspected, serial dilution of the sample should be performed to exclude falsely low prolactin readings, which could otherwise lead to underestimation of a true prolactinoma and misclassification of the aetiology [9].

From a management perspective, endoscopic transsphenoidal resection remains the primary treatment for silent somatotroph adenomas presenting with mass-effect symptoms [10]. As in this case, cavernous sinus involvement typically precludes complete resection, and adjuvant radiotherapy or close surveillance is required for residual disease. Postoperative complications, including transient diabetes insipidus and adrenal insufficiency, are well-recognised following pituitary surgery and should be anticipated and managed proactively. Integrated pituitary MDT decision-making, incorporating neurosurgical, endocrine, radiological, and neuropathological expertise, is essential for optimal outcomes.

This case report has some limitations. An OGTT to assess GH suppression was not performed because urgent neurosurgical intervention was prioritised due to the patient's progressive neurological compromise. Consequently, biological GH secretory activity could not be completely characterised, and this case should be interpreted within these limitations. 

## Conclusions

Silent somatotroph adenomas present a diagnostic paradox: acromegaloid phenotypic features can occur in the complete absence of biochemical GH excess. IGF-1 alone is an insufficient diagnostic tool in this setting, and clinicians evaluating mass-effect pituitary lesions with acromegaloid features should proactively pursue histopathological assessment even when biochemistry appears reassuring. Immunohistochemistry for GH and Pit-1 should be performed routinely on all resected pituitary tumours. Long-term combined endocrine and neurosurgical follow-up is strongly recommended, given the potential for biochemical progression and the aggressive natural history of silent somatotroph PitNETs.
